# RNA recording in single bacterial cells using reprogrammed tracrRNAs

**DOI:** 10.1038/s41587-022-01604-8

**Published:** 2023-01-05

**Authors:** Chunlei Jiao, Claas Reckstadt, Fabian König, Christina Homberger, Jiaqi Yu, Jörg Vogel, Alexander J. Westermann, Cynthia M. Sharma, Chase L. Beisel

**Affiliations:** 1 Helmholtz Institute for RNA-based Infection Research (HIRI), Helmholtz Centre for Infection Research (HZI), Würzburg, Germany; 2 Department of Molecular Infection Biology II, Institute of Molecular Infection Biology, University of Würzburg, Würzburg, Germany; 3 Institute of Molecular Infection Biology, University of Würzburg, Würzburg, Germany; 4 Medical Faculty, University of Würzburg, Würzburg, Germany

**Keywords:** Expression systems, Transcriptomics

## Abstract

Capturing an individual cell’s transcriptional history is a challenge exacerbated by the functional heterogeneity of cellular communities. Here, we leverage reprogrammed tracrRNAs (Rptrs) to record selected cellular transcripts as stored DNA edits in single living bacterial cells. Rptrs are designed to base pair with sensed transcripts, converting them into guide RNAs. The guide RNAs then direct a Cas9 base editor to target an introduced DNA target. The extent of base editing can then be read in the future by sequencing. We use this approach, called TIGER (transcribed RNAs inferred by genetically encoded records), to record heterologous and endogenous transcripts in individual bacterial cells. TIGER can quantify relative expression, distinguish single-nucleotide differences, record multiple transcripts simultaneously and read out single-cell phenomena. We further apply TIGER to record metabolic bet hedging and antibiotic resistance mobilization in *Escherichia coli* as well as host cell invasion by *Salmonella*. Through RNA recording, TIGER connects current cellular states with past transcriptional states to decipher complex cellular responses in single cells.

## Abstract (web summary)

RNA transcripts in bacteria are recorded as DNA modifications using a base editor.

## Main

The identity and behavior of a cell depend not only on its current intracellular make-up and extracellular environment, but also on its past states. Past states determine the trajectory of a cell and shape the cell’s future physiology and functions, such as in development, aging, carcinogenesis, bet hedging and synthetic multistable systems^1–5^. Past states can also reflect key events that are no longer detectable, such as a host that was previously infected by a virus, a bacterial pathogen that transited through different tissues or a cell temporarily tolerant to antibiotics^6,7^. Currently, defining a cell’s state most frequently involves measuring its transcriptional profile. Accordingly, numerous techniques have been developed that can determine the identity and abundance of RNA transcript levels as well as whether the abundances are actively changing^8–10^. Measurements can even be performed in individual cells, revealing distinct cellular programs within an isogenic population in a homogenous environment^11–16^. A central limitation of these techniques, however, is that they can only capture the current state of a cell. At most, previous states can be approximated by measuring asynchronous cells over time coupled with computational prediction tools^17^. Recording past RNA transcripts instead offers a direct means of surveying the past as well as the present. RNA recording so far has been achieved by converting randomly captured RNAs into preserved DNA spacers through CRISPR acquisition^18,19^. However, this approach requires sequencing of massive populations of cells, masking single-cell processes^20–22^.

Here, we introduce TIGER (transcribed RNAs inferred by genetically encoded records), a technique for recording the presence and abundance of RNAs of interest in individual bacterial cells. Using TIGER, we record messenger RNAs (mRNAs) and small RNAs (sRNAs) in different bacteria, with single-nucleotide precision, quantification of relative transcript levels, recording of multiple transcripts and single-cell resolution. We further apply TIGER to record metabolic bet hedging, mobilization of antibiotic resistance and a bacterial pathogen infecting a host cell. TIGER thus opens user-selected transcripts in the past to single-cell interrogation.

## Results

### Recording cellular RNAs with TIGER

**Fig. 1:**
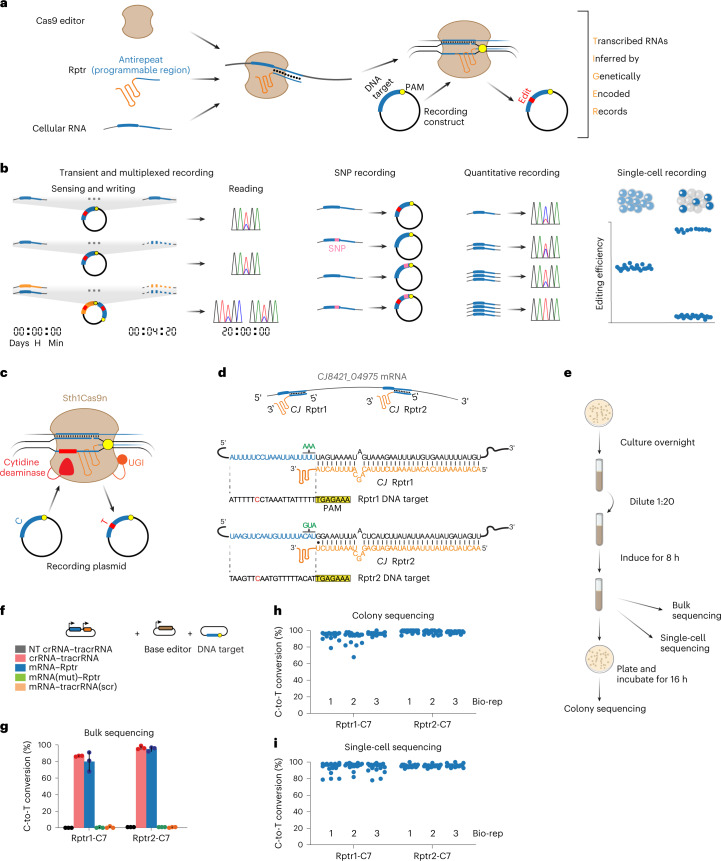
Programmable DNA recording of cellular RNAs with TIGER. **a**, Overview of TIGER. A designed Rptr pairs within the selected transcript to form a gRNA that can be used by the Cas editor. The editor is then directed to a matching DNA sequence, creating a permanent edit only in the presence of the transcript. Within the transcript, the thin blue line is the region that pairs with the Rptr, whereas the thick blue line is the resulting guide sequence that specifies the DNA target. The PAM is depicted in yellow. **b**, Recording capabilities offered by TIGER. The single-cell recording captures phenomena observable only at the level of individual cells. **c**, Using the Sth1Cas9n cytosine base editor for RNA recording. Recording is assessed via the conversion of a C in the editing window (marked as red) to a T based on relative peak heights in Sanger sequencing chromatographs. UGI, uracil-DNA glycosylase inhibitor. **d**, Designed Rptr–DNA target pairs for sensing the *CJ8421_04975* mRNA. Green nucleotides designate mutations made to disrupt DNA targeting. **e**, Experimental process to record the *CJ8421_04975* mRNA constitutively expressed in *E. coli*. The recording can be read out through bulk sequencing, colony sequencing and single-cell sequencing. **f**, Constructs for assessing RNA recording. mRNA(mut), three nucleotides mutated at the 3′ end of the resulting gRNA guide. tracrRNA(scr), tracrRNA with the antirepeat domain scrambled. **g**, Results from bulk sequencing. Colors correspond to those in **f**. **h**, Results from colony sequencing. **i**, Results from single-cell sequencing. See the representative plots and gates associated with single-cell DNA sequencing in Extended Data Fig. 2a. C7 in **g**–**i** designates the C in the target assessed for C-to-T conversion. Values in **g**–**i** represent the mean and standard deviation of independent experiments starting from three separate colonies. Each dot for colony sequencing or single-cell DNA sequencing represents one of 20 sequenced colonies or single cells in the biological replicate. Bio-reps, biological replicates starting from separate colonies. Source data

To record selected RNA transcripts in living cells, we leveraged our previous discovery of cellular RNAs being converted into guide RNAs (gRNAs) that direct DNA cleavage by Cas9 (ref. 23). The *trans*-activating CRISPR RNA (tracrRNA), an RNA processing factor in many CRISPR–Cas immune systems^24^, is responsible for this conversion by hybridizing with a cellular RNA to form an imperfect duplex recognized by Cas9. By engineering reprogrammed tracrRNAs (Rptrs) that base pair with an RNA of interest, we and others could convert the hybridizing portion of this RNA into a gRNA^23,25^. An introduced DNA target with a compatible protospacer-adjacent motif (PAM) could then be monitored, with target binding or cleavage indicating the presence of the associated RNA. While we previously applied Rptrs for multiplexed RNA detection as part of an in vitro RNA detection approach called LEOPARD^23^, we reasoned that this same platform could be implemented in cells to record the presence of different RNAs of interest in vivo (Fig. 1a).

To establish this approach, we introduce DNA encoding three components: a designed Rptr, a corresponding target DNA sequence and a Cas9 editor. The expressed Rptr pairs with the RNA of interest, resulting in the formation of a gRNA bound by Cas9. The gRNA then directs Cas9 to the DNA target encoded on a multi-copy plasmid, resulting in a precise and permanent edit that is passed to future progeny and can be read at a later point in time. Because each DNA target is unique to the sensed RNA of interest, multiple Rptrs and corresponding targets can be introduced into the same cell to allow scalable multiplexing of RNA recording. The recording occurs in each cell and could be rendered quantitative by introducing multiple copies of each DNA target (for example, on a multi-copy plasmid) (Fig. 1b). We call this platform TIGER as the in vivo parallel to our detection platform LEOPARD.

### Recording heterologous transcripts in *E. coli*

**Extended Data Fig. 1:**
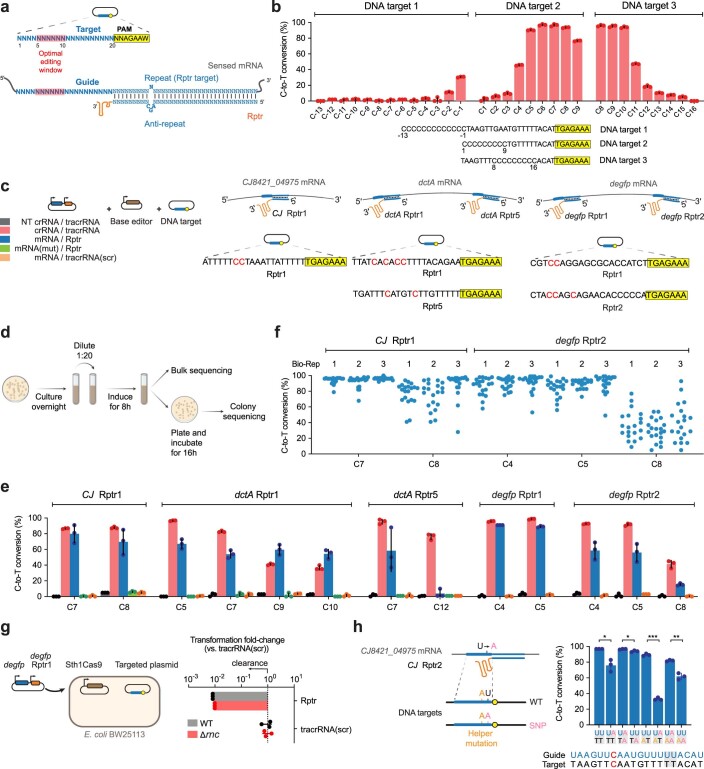
Establishing and optimizing TIGER in *E. coli*. (**a**) Design rules for a Rptr and DNA target for the Sth1Cas9n cytosine base editor. The PAM is boxed in yellow. (**b**) Defining the optimal editing window for Sth1Cas9n cytosine base editor. Three sgRNAs are designed with poly Cs located in different positions of the guide sequence. Letter-number pairs (for example, C8) indicate the base monitored to quantify C-to-T conversion. (**c**) Recording three different heterologous transcripts using TIGER. The left indicates the coloring scheme used in **e** and **f**. (**d**) Experimental setup for bulk sequencing and colony sequencing in *E. coli*. (**e**) Bulk sequencing of the recorded transcripts. (**f**) Colony sequencing of the recorded transcripts. (**g**) Negligible contribution of endogenous RNase III to Rptr activity. Activity was assessed based on plasmid interference by Sth1Cas9. Left: experimental setup. Right: transformation fold-change comparing the designated Rptr to the scrambled tracrRNA. (**h**) SNP detection through the use of helper mutations. Values in **b**, **e**, **g**, and **h** represent the mean and standard deviation of independent experiments starting from three separate colonies. Each dot for colony sequencing in f represents one sequenced colony. *: p < 0.05, **: p < 0.01, ***: p < 0.001, n.s.: p > 0.05. P-values were calculated using a two-tailed Student’s t-test with unequal variance. Source data

As a proof-of-concept, we attempted to record the presence of a heterologous transcript that is constitutively expressed. To generate a detectable edit, we used a Cas9 cytosine base editor that uses rAPOBEC1 to convert any C into a T within an editing window in the target^26^ (Fig. 1c and Extended Data Fig. 1a). The Cas9 comes from the CRISPR1 locus of *Streptococcus thermophilus* (Sth1Cas9), where this nuclease exhibited the most consistent activity with Rptrs in our previous work^23^. Sth1Cas9 also requires a strict PAM (5′-NNAGAAW-3′)^27,28^, minimizing the chance of editing the sensed transcript’s own gene. Similar to previous platforms that record environmental signals^29–31^, the converted T could be read at a later time through Sanger sequencing or next-generation sequencing. As a test case, the heterologous transcript encoded by the *CJ8421_04975* gene from *Campylobacter jejuni* CG84-21 was constitutively expressed in *E. coli* along with one of two designed Rptrs (Fig. 1d). The base editor was inducibly expressed to control the period of recording. Each Rptr base pairs with a distinct location in the transcript containing at least one C in the delineated six-nucleotide editing window (Extended Data Fig. 1a,b). The DNA target flanked by a recognized PAM was encoded on a low-copy plasmid (roughly five copies per cell) to enable measurement by sequencing. Using this setup, we had the opportunity to quantify recording in three ways: isolating plasmid DNA from the culture and sequencing the DNA target, plating the culture and sequencing the DNA target amplified from individual colonies, or sorting individual cells from the culture and sequencing the amplified DNA target (Fig. 1e). Apart from single-cell sequencing, colony sequencing would approximate recording at the single-cell level because each colony grows from a single cell.

**Extended Data Fig. 2:**
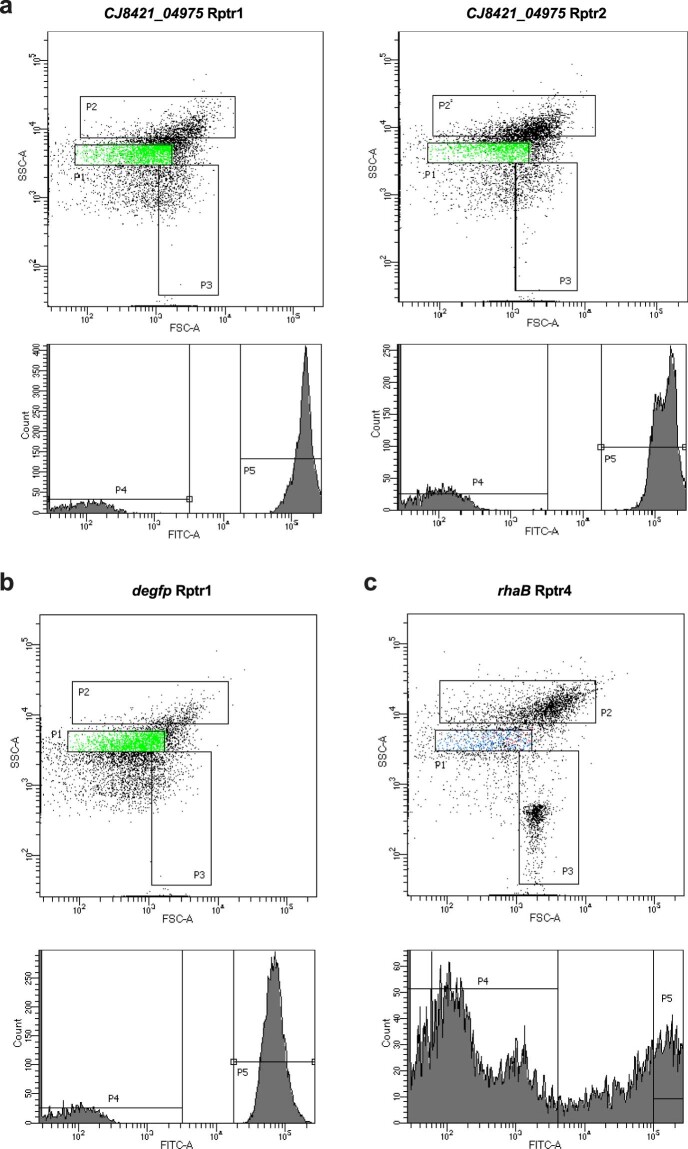
Gating strategy for single-cell DNA sequencing or FACS-based colony sequencing. (**a**) Representative plots and gates associated with single-cell DNA sequencing using constitutively expressed *CJ8421_04975* and its two Rptrs in Fig. 1i. (**b**) Representative plots and gates associated with single-cell DNA sequencing using constitutively expressed *degfp* in Fig. 2h. (**c**) Representative plots and gates associated with FACS-based colony sequencing using inducible *rhaB* and its Rptr in Fig. 3g. A side scatter (SSC-A) vs. forward scatter (FSC-A) density plot was used to exclude doublets (P2) and debris (P3) to get the singlets (P1). In **a** and **b**, two subsets, P4 and P5, were generated from P1 based on fluorescent intensity in the FITC channel. P4 referred to events with background fluorescence, P5 referred to events with high fluorescence. Cells sorted from P5 were used for single-cell DNA sequencing. In **c**, P4 referred to >99% detected events in the L-rhamnose-free control sample (0 mM L-rhamnose). P5 gates for events with fluorescence intensities above 10^5^. Cells from P4 and P5 at 3.33 mM L-rhamnose were sorted into two separate tubes before plating.

For each designed Rptr and the corresponding DNA target, bulk sequencing yielded nearly complete editing within the target, similar to a designed CRISPR RNA (crRNA)–tracrRNA pair against the same target (Fig. 1f,g and Extended Data Fig. 1c–e). In contrast, editing was negligible when mutating the targeting portion within the transcript (mRNA(mut)) or scrambling the base-pairing portion of the Rptr (tracrRNA(scr)). Similarly high levels of editing were observed for every sequenced colony (Fig. 1h and Extended Data Fig. 1f) that each grew from a single cell or for every sequenced cell isolated by fluorescence-activated cell sorting (FACS) (Fig. 1i and Extended Data Fig. 2a). We also tested additional heterologous transcripts, finding that TIGER could record the presence of the *dctA* transcript from *C. jejuni* and a transcript encoding green fluorescent protein (*gfp*) (Extended Data Fig. 1c–e). Recording did not require the processing factor RNase III (Extended Data Fig. 1g), lending to the broad use of TIGER. These results show that TIGER can be used to record the presence of different RNA transcripts, even in single cells.

**Fig. 2:**
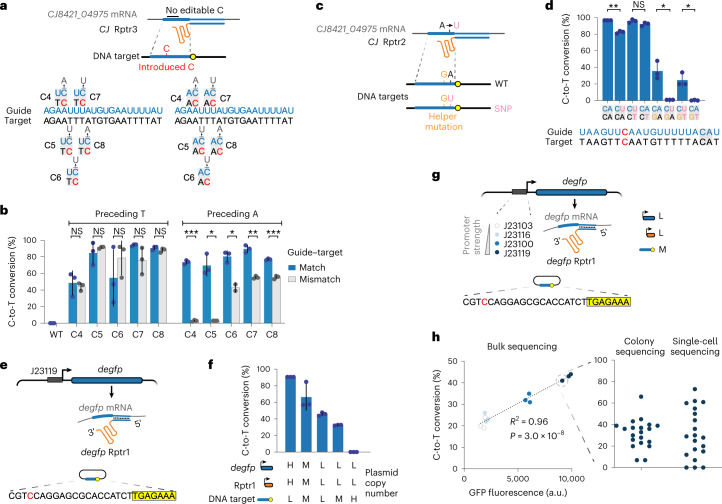
Expanding TIGER for extended target selection, SNP detection and quantitative recording. **a**, Using DNA targets lacking a C in the editing window. A representative guide derived from the *CJ8421_04975* mRNA was paired with *CJ* Rptr3 along with the corresponding DNA target. At different locations within the target, a C was swapped into the target along with a preceding favored T or disfavored A (if not already present). The guide portion of the *CJ8421_04975* transcript was mutated so there are no mismatches (match) or a single mismatch (mismatch) at the introduced C. **b**, Bulk sequencing of the different guide–DNA target pairs (Fig. 1e). **c**, Enhancing SNP detection through the use of helper mutations. The SNP (pink) was swapped into position 19 of the guide portion of the *CJ8421_04975* transcript. A helper mutation (orange) was also added at position 18 of the guide. **d**, Bulk sequencing of *CJ8421_04975* transcript recording with the different guide–target mutations. The swapped bases are within the gray box. **e**, Recording the constitutively expressed *degfp* transcript. **f**, Bulk sequencing of *degfp* transcript recording when placing the TIGER machinery on plasmids with different copy numbers. H, high-copy; M, medium-copy and L, low-copy. **g**, Assessing quantitative recording by varying the promoter strength of *degfp*. **h**, Correlating promoter strength and recording efficiency. The left shows bulk sequencing. GFP fluorescence was measured by flow cytometry analysis. See **g** for the corresponding colors. The *P* value was calculated using linear regression analysis, *n* = 12. The right shows colony sequencing and single-cell sequencing from one culture with the strongest J23119 promoter. a.u., arbitrary units. Values in **b**, **d** and **f** represent the mean and standard deviation of independent experiments starting from three separate colonies. Each dot for colony sequencing or single-cell DNA sequencing represents one of 20 sequenced colonies or single cells in the biological replicate. The dot plot is representative of independent experiments starting from three separate colonies. See the representative plots and gates associated with single-cell DNA sequencing in Extended Data Fig. 2b. *P < 0.05, **P < 0.01, ***P < 0.001, NS (not significant) *P* > 0.05. *P* values were calculated using a two-tailed Student’s *t*-test with unequal variance. Source data

Applying TIGER with base editing requires a C within the editing window of the target. While 82% of random RNA with six nucleotides should contain at least one editable C, nucleotide bias in certain sequences, transcripts and organisms could reduce this frequency. One workaround is introducing a C into the target (Fig. 2a), although this creates a gRNA–target mismatch that could impair editing. Despite this concern, we found that an introduced mismatch did not significantly affect editing if the C was immediately downstream of a T (*P* = 0.18–0.67), the preferred context for the rAPOBEC1 cytidine deaminase domain of the base editor^32^ (Fig. 2b). If the C was immediately downstream of an A, a less preferred context for the rAPOBEC1 domain, editing was significantly reduced (*P* = 8 × 10^−5^–0.01) but still high when the C was placed at some positions in the target (Fig. 2b). This general workaround expands the range of RNA sequences that can be sensed using TIGER.

### Quantitative RNA recording with single-nucleotide resolution

Beyond recording the presence of a distinct transcript, distinguishing sequences that differ by a single nucleotide could enable the detection of single-nucleotide polymorphisms (SNPs) such as those associated with antibiotic resistance, emergent viral variants, or cancer progression^33–36^. The SNP could also represent an instance of transient RNA editing^37,38^. The SNP alone was not sufficient to yield large differences in editing for the wild-type (WT) and SNP-containing RNAs (Fig. 2c,d). However, introducing a ‘helper’ mutation next to the SNP in the target eliminated editing for the mismatched pairs (for example, SNP RNA–WT target) while only partially reducing editing for the matched pairs (for example, SNP RNA–SNP target) (Fig. 2d). The position of the SNP in the target was important, as the editing difference between the WT and SNP RNAs was reduced when the point mutation was farther from the PAM-proximal end of the target (Extended Data Fig. 1h).

Moreover, quantifying relative transcript levels could enable the measurement of changes in transcriptional patterns, such as when distinguishing different tissue types or determining the surrounding environments experienced by a cell. As a first attempt, we varied the copy number of the plasmid encoding a sensed fluorescent reporter deGFP^39^ (Fig. 2e), which yielded the expected editing improvement with a higher copy number (Fig. 2f). Varying the copy number of the DNA target plasmids had the reverse effect (Fig. 2f), in line with more targets being available to edit. As a more quantitative approach, we inserted constitutive promoters of four different strengths upstream of *degfp* and measured the correlation between cellular fluorescence and recording (Fig. 2g). For the medium-copy DNA target plasmid, we observed a strong and direct correlation (*R*^2^ = 0.96) for bulk sequencing (Fig. 2h). Colony sequencing and single-cell sequencing with the strongest *degfp* promoter revealed widespread variability in editing (Fig. 2h and Extended Data Fig. 2b), potentially reflecting stochasticity in gene expression indicative of single-cell phenomena or variability in segregation of the recording plasmid. TIGER therefore can be implemented to distinguish a single point mutation and can quantify relative transcript levels.

### Mitigating the impact of TIGER on the sensed transcript

**Extended Data Fig. 3:**
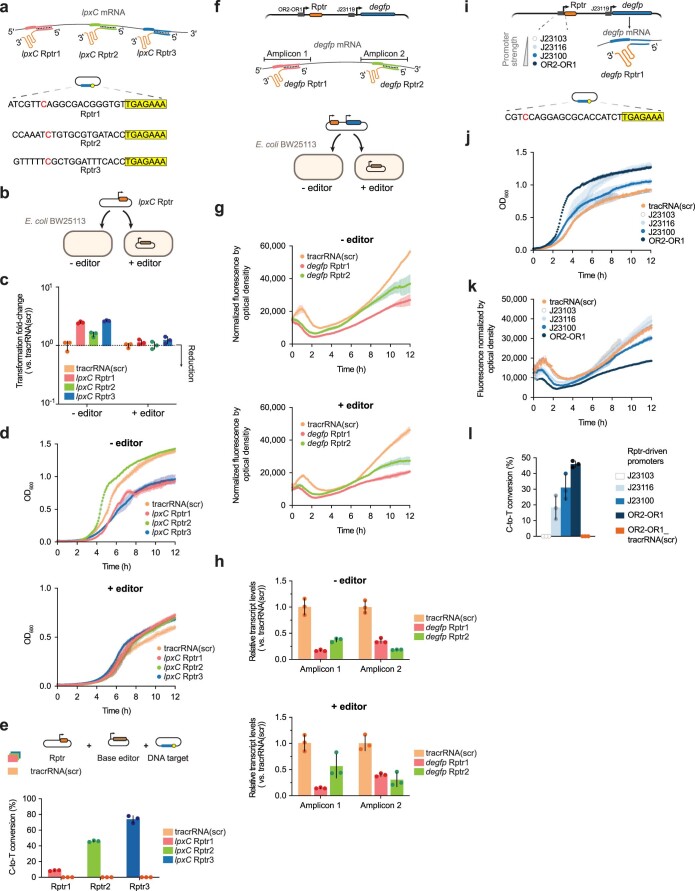
Impact of TIGER on sensed transcripts. (**a**) Recording the essential transcript *lpxC* in *E. coli*. (**b**) Measuring the impact of *lpxC* Rptr expression in the presence or absence of the base editor. (**c**) Impact on colony-forming units following transformation of the Rptr plasmid. (**d**) Impact on culture turbidity after inoculating transformed colonies. (**e**) Bulk sequencing of *lpxC* mRNA recording. Colors in **b**, **d** and **e** correspond to the Rptrs shown in a, with orange indicating the scrambled tracrRNA (tracrRNA(scr)). In **c - e**, *lpxC* Rptrs were expressed from a high-copy plasmid, with their targets in a low-copy plasmid. (**f**) Assessing the impact of TIGER on deGFP when recording its expression in *E. coli*. Two *degfp* Rptrs were tested in strains with or without the base editor. (**g**) Impact on deGFP fluorescence during growth in a microplate reader. (**h**) Impact on *degfp* transcript levels as determined by RT-qPCR. Two different amplicons covering the Rptr target sites (amplicon 1 for Rptr1, amplicon 2 for Rptr2) within the *degfp* transcript were selected for relative RNA quantification. Colors in **g** and **h** correspond to the Rptrs shown in **f**, with orange indicating the scrambled tracrRNA (tracrRNA(scr)). (**i**) Varying the promoter strength driving *degfp* Rptr expression. (**j**) Impact on culture turbidity during growth in a microplate reader. (**k**) Impact on deGFP fluorescence during growth in a microplate reader. (**l**) Impact on transcript recording via bulk sequencing. Colors in **j**–**l** correspond to the promoter strengths in **i**, while orange represents the scrambled tracrRNA driven by the strongest OR2-OR1 constitutive promoter. In **g**, **h**, **j**, **k**, and **l**, the *degfp* Rptr along with the *degfp* mRNA were expressed from a low-copy plasmid, while the target was encoded in a medium-copy plasmid. Values in **b**, **d**, **e**, **g**, **h**, **j**, **k**, and **l** represent the mean (bar or dot) and standard deviation (error bar or shaded region) of independent experiments starting from three separate colonies. Source data

The impact of varying the copy number of the TIGER machinery raised the question: how does TIGER affect the abundance and translation of sensed transcripts? Ideally, sensing a transcript would have no impact on its expression levels and function, although the mechanism of regulation—using a fragment of the transcript as a gRNA bound by Cas9—should have some impact. We began by recording the essential *lpxC* (*envA*) transcript^40^ (Extended Data Fig. 3a), where a strong negative effect would affect cell viability and fitness. Testing three distinct Rptrs, we did not observe any reduction in colony counts when transforming the final Rptr plasmid (Extended Data Fig. 3b,c) and only observed modestly reduced growth in liquid culture for two of the Rptrs in the absence of the base editor (Extended Data Fig. 3d). All three Rptrs yielded measurable recording by bulk sequencing (Extended Data Fig. 3e), demonstrating that TIGER can record essential transcripts.

To directly quantify the impact on the sensed transcript, we assessed two Rptrs against the *degfp* transcript (Extended Data Fig. 3f). Both reduced deGFP levels by two- to fourfold based on cell fluorescence and quantitative PCR with reverse transcription (RT–qPCR) (Extended Data Fig. 3g,h). As the reduction is likely due to the direct interaction with the Rptr, we reasoned that reducing Rptr expression could mitigate this effect. Accordingly, using progressively weaker constitutive promoters to drive Rptr expression abrogated the impact on deGFP levels (Extended Data Fig. 3i–k). While the extent of editing by bulk sequencing also decreased from roughly 46% with the strongest promoter, a weaker promoter still yielded moderate editing (roughly 18%) and no detectable decrease in deGFP levels based on cell fluorescence (Extended Data Fig. 3l). Overall, Rptrs can affect the sensed transcript, although reducing Rptr expression can reduce the impact albeit with a concomitant drop in the recording efficiency.

### RNA recording of metabolic bet hedging

Many processes are typified by transcripts that are differentially expressed within the cell population, giving rise to expression heterogeneity that is undetectable by traditional bulk approaches yet essential for multicellular programs or survival in uncertain and changing environments^20–22^. To explore whether TIGER is able to capture expression heterogeneity, we focused on sugar catabolism in *E. coli*. Most catabolic pathways are induced only in the presence of the corresponding sugar substrate, preserving resources needed to produce the transport and catabolic machinery. When supplied at subsaturating concentrations, many sugars fully induce expression of their dedicated multi-gene catabolic pathway, but only in a fraction of the cellular population^41^. This heterogeneous response is thought to represent a bet-hedging strategy in fluctuating environments as well as a means of distributing the taxing work of sugar catabolism when multiple sugars are present^4^.

**Fig. 3:**
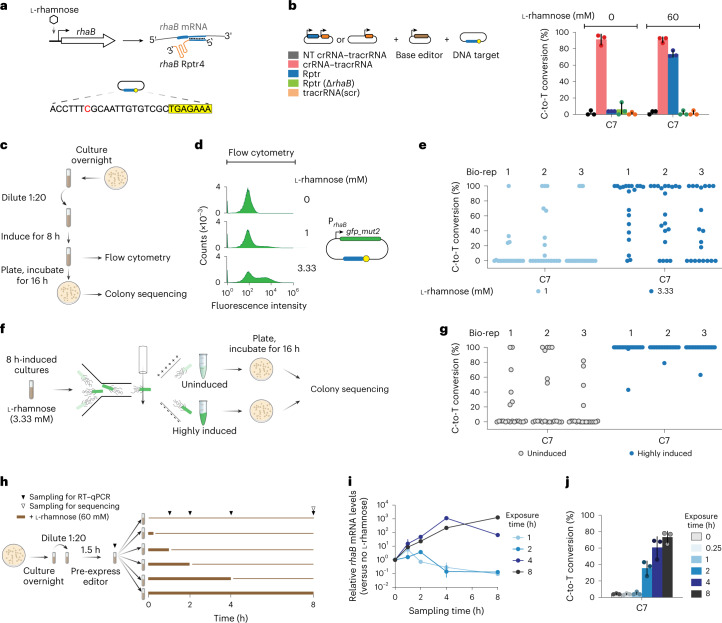
Recording of endogenous, transient single-cell programs. **a**, Recording of endogenous *rhaB* expression induced by the sugar l-rhamnose in *E. coli*. **b**, Bulk sequencing of *rhaB* transcript recording in the presence or absence of l-rhamnose. *∆rhaB*: deletion of the *rhaB* gene. The crRNA controls were expressed from the constitutive J23119 promoter, explaining efficient recording in the presence or absence of l-rhamnose. **c**, Experimental process to record the *rhaB* mRNA in *E. coli* via colony sequencing and measure single-cell induction of a transcriptional reporter. **d**, Bimodal, dose-dependent induction of *rhaB* expression using the transcriptional reporter. Reporter levels were measured by flow cytometry analysis. The histograms are representative of independent experiments starting from three separate colonies. **e**, Colony sequencing of *rhaB* mRNA recording with cells cultured in different concentrations of l-rhamnose. **f**, Experimental process to connect transcriptional reporter induction with the recording efficiency via FACS. **g**, Colony sequencing of *rhaB* mRNA recording following FACS. See the representative plots and gates associated with FACS-based colony sequencing in Extended Data Fig. 2c. Each dot in **e** and **g** represents one of 20 sequenced colonies in a biological replicate. **h**, Experimental process in **i** and **j** to assess recording of the *rhaB* mRNA following dosing of l-rhamnose for different lengths of time. **i**, Measured *rhaB* mRNA levels as determined by RT–qPCR. Levels are relative to cells cultured in the absence of l-rhamnose for the same period of time. **j**, Bulk sequencing of *rhaB* mRNA recording with different exposure times to l-rhamnose. Linear regression of the editing frequency between 1 and 8 h and editing efficiency yielded *R*^2^ = 0.74, *P* = 3.1 × 10^−4^. Values in **b**, **i** and **j** represent the mean and standard deviation of independent experiments starting from three separate colonies. Source data

**Extended Data Fig. 4:**
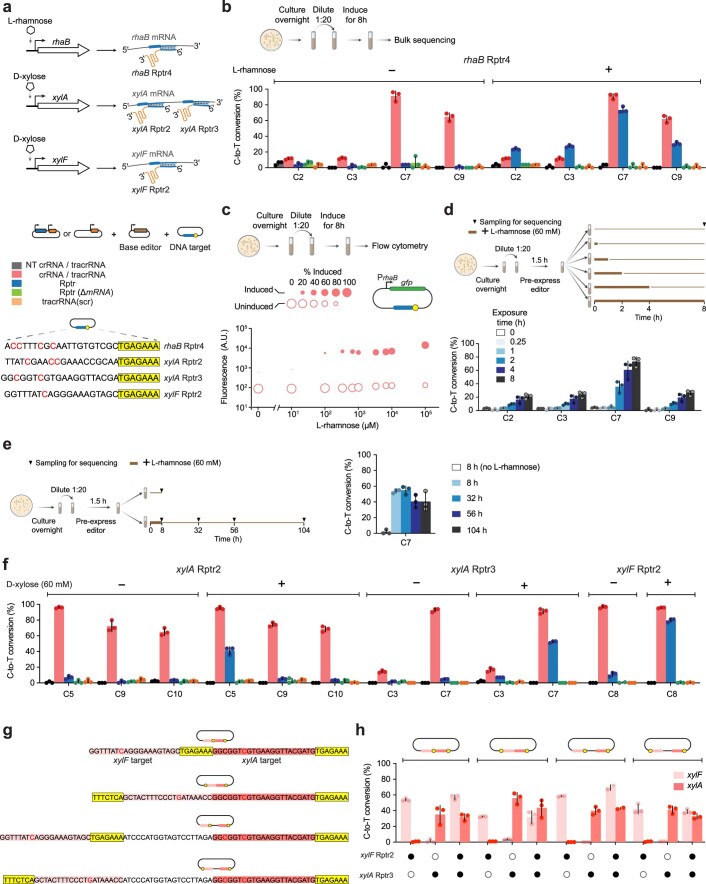
Multiplexed recording of endogenous transcripts from sugar catabolic pathways. (**a**) Recording the *rhaB*, *xylA* and *xylF* transcripts in *E. coli*. The expression of each gene is induced by the indicated sugar. The left indicates the coloring scheme used in **b** and **f**. (**b**) Bulk sequencing of recorded *rhaB* expression induced by L-rhamnose. (**c**) Detecting L-rhamnose-induced population heterogeneity by flow cytometry analysis with a fluorescent transcriptional reporter. Each dot represents the mean of independent experiments starting from three separate colonies. The area of each dot scales with the fraction of cells in that population. (**d**) and (**e**) Recording of *rhaB* expression transiently induced with L-rhamnose. (**f**) Bulk sequencing of recorded *xylA and xylF* expression induced by D-xylose. See a for the bar coloring. Expression of the crRNA controls was driven by a constitutive promoter J23119, where editing is expected even in the absence of D-xylose. (**g**) Constructs for multiplexed recording of *xylA and xylF* expression. The DNA targets are encoded in different configurations and are spaced by different distances. (**h**) Multiplexed recording of *xylA* and *xylF* expression in the presence of D-xylose. Solid dot, presence of the Rptr; hollow dot, absence of the Rptr. Values in **b**, **d**, **e**, **f** and **h** represent the mean and standard deviation of independent experiments starting from three separate colonies. Source data

We selected l-rhamnose as a representative sugar because it induces bimodal expression of dedicated catabolic and transporter genes^41^. We focused on recording the *rhaB* transcript encoding one catabolic enzyme in l-rhamnose assimilation (Fig. 3a)^42^. Using bulk sequencing, we observed negligible editing in the absence of l-rhamnose and 74% editing in the presence of l-rhamnose (Fig. 3b and Extended Data Fig. 4a,b). As expected, deleting the chromosomal copy of *rhaB* or scrambling the base-pairing portion of the Rptr yielded negligible editing even in the presence of l-rhamnose (Fig. 3b and Extended Data Fig. 4a,b). Turning to the single-cell level, we confirmed that l-rhamnose induces bimodal expression of *rhaB* using a fluorescent transcriptional reporter, where the fraction of induced cells increased with higher l-rhamnose concentrations (28% induced for 1 mM, 48% induced for 3.33 mM) (Fig. 3c,d and Extended Data Fig. 4c). Under these same growth conditions, editing from colony sequencing yielded a bimodal distribution that paralleled the distributions with the fluorescent reporter (23% edited for 1 mM, 75% edited for 3.33 mM) (Fig. 3e).

To assess whether induction and editing were directly linked, we sorted high- and low-fluorescence cells cultured with 3.33 mM l-rhamnose followed by colony sequencing (Fig. 3f). Doing so yielded complete editing for virtually all colonies from the high-fluorescence population and no editing for most colonies from the low-fluorescence population (Fig. 3g and Extended Data Fig. 2c). Some colonies from the low-fluorescence population yielded variable extents editing, suggesting that the cells were transitioning into or out of the induced state. These results demonstrate that TIGER can record single-cell phenomena.

### Transient and multiplexed RNA recording

**Fig. 4:**
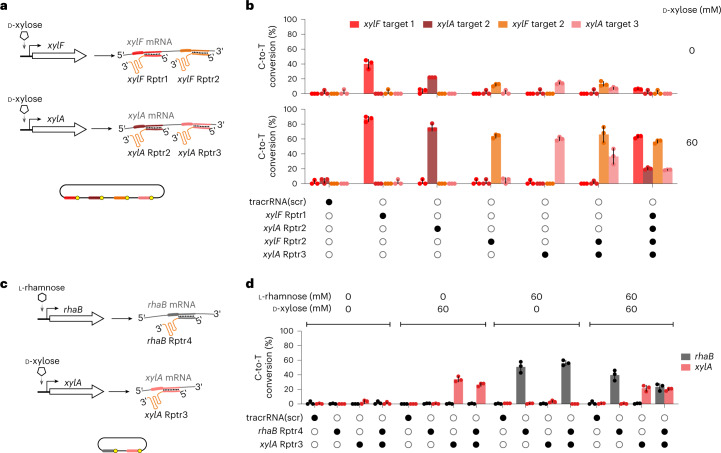
Multiplexed recording of endogenous RNAs. **a**, Multiplexed recording of *xylF* and *xylA* expression induced by d-xylose in *E. coli*. The recording plasmid encoded four DNA targets matched to *xylF* and *xylA*, with adjacent targets separated by 20 bps. **b**, Bulk sequencing of multiplexed *xylF* and *xylA* mRNA recording following exposure to d-xylose for 8 h. **c**, Multiplexed recording of *rhaB* expression and *xylA* expression, respectively, induced by l-rhamonse and d-xylose in *E. coli*. The recording plasmid encoded two DNA targets matching *rhaB* and *xylA*, with adjacent targets separated by 20 bps. **d**, Bulk sequencing of multiplexed *rhaB* and *xylA* mRNA recording following exposure to d-xylose and/or l-rhamnose for 8 hours. Values in **b** and **d** represent the mean and standard deviation of independent experiments starting from three separate colonies. Solid dot, presence of the Rptr; Hollow dot, absence of the Rptr. Source data

Beyond recording single-cell phenomena, sugar catabolism presented additional opportunities to test the capabilities offered by TIGER. As some processes depend on the duration of expression^43^, we evaluated how the timing of transcript expression affects recording. By including l-rhamnose in the culture media for different durations (Fig. 3h), we found that the editing frequency correlated linearly with the induction time (*R*^2^ = 0.74 between 1 and 8 h to account for the delay in the induction of *rhaB*) (Fig. 3i,j and Extended Data Fig. 4d). Furthermore, the extent of editing was maintained even after culturing the cells up to four subsequent days in the absence of l-rhamnose (Extended Data Fig. 4e). Some processes also regulate multiple signaling pathways, underscoring the need for multiplexed recording. In this case, TIGER can be readily scaled by expressing multiple Rptrs and encoding the associated DNA targets in the same locus. To explore multiplexed recording, we incorporated the sugar d-xylose that also induces expression of dedicated catabolic and transport genes (Fig. 4a). Four Rptrs against the *xylA* and *xylF* transcripts yielded measurable editing of adjacent *xylA-xylF* targets by bulk sequencing, but only in the presence of d-xylose (Fig. 4b and Extended Data Fig. 4f–h). The extent of recording decreased for multiplexed versus single-plex recording, suggesting limits to large-scale recording in single cells. Separately, recording the *xylA* and *rhaB* transcripts simultaneously yielded editing only in the presence of the cognate sugar (Fig. 4c,d). TIGER therefore can provide insights into the duration of past events while recording the presence of multiple transcripts from coordinated and orthogonal pathways.

### Recording expression of mobilized antibiotic resistance

After establishing the capabilities of TIGER, we aimed to apply this technique to two areas of application: recording a nonnative transcript that enters the cell during the recording period, and recording changes in endogenous transcript levels on key cellular events. Within the first broad category, the entering transcript could represent an invading virus or another mobile genetic element, a transformed genetic construct or an administered gene therapy. One important example in this area is the spread of antibiotic resistance through a microbial population. Antibiotic resistance is a growing cause of death globally^33^, and remains an urgent threat. Resistance is often passed through mobile genetic elements such as conjugative plasmids that can be actively passed between otherwise unrelated bacteria. Recording which cells receive this resistance, whether they maintain or quickly lose resistance, could provide insights into the routes of dissemination and how these routes can be mitigated.

**Fig. 5:**
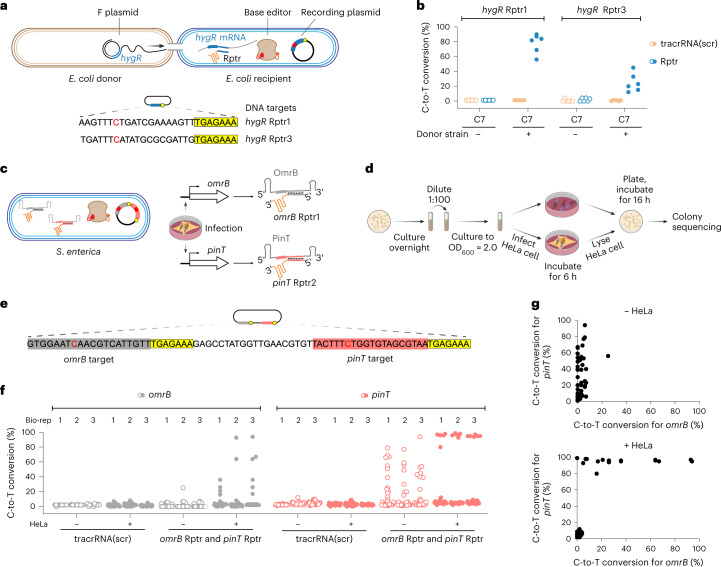
Recording of mobilized antibiotic resistance and infection-induced sRNAs. **a**, Recording of *hygR* expression transferred by a conjugative plasmid between *E. coli* strains. two distinct Rptrs to record *hygR* expression in the recipient strain. **b**, Colony sequencing of *hygR* mRNA recording following tranconjugation. Recording was measured in transconjugants (+Donor strain) or unconjugated recipients (−Donor strain). Each dot for colony sequencing represents one of the six sequenced colonies. Results are representative of duplicate independent experiments. **c**, Recording the infection-induced sRNAs OmrB and PinT in intracellular *Salmonella*. *Salmonella* cells are equipped with the TIGER machinery, including a medium-copy recording plasmid encoding adjacent DNA targets for OmrB and PinT. **d**, Experimental process for **f** and **g** to record the sRNAs following invasion of HeLa cells or growth in the cell culture medium. HeLa cells were infected with *Salmonella* at a multiplicity of infection of 10 followed by gentamicin selection to remove extracellular *Salmonella* cells. **e**, DNA construct for recording OmrB and PinT. **f**, Colony sequencing of multiplexed OmrB and PinT recording. **g**, Recording coexpression of OmrB and PinT from colony sequencing. The dots from all three biological replicates in **f** are included. Values in **b**, **f** and **g** represent the mean and standard deviation of independent experiments starting from three separate colonies. Each dot in **f** and **g** represents one of 30 sequenced colonies in a biological replicate. Source data

**Extended Data Fig. 5:**
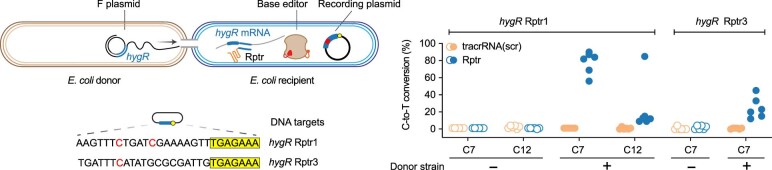
Recording of mobilized antibiotic resistance. Recording of *hygR* expression transferred by a conjugative plasmid between *E. coli* strains. Left: two distinct Rptrs to record *hygR* expression in the recipient strain. Right: Recording in transconjugants (+ Donor strain) or unconjugated recipients (- Donor strain) using a designed Rptr or a scrambled tracrRNA. Each dot for colony sequencing represents one sequenced colony. Source data

We therefore applied TIGER to record a conjugative plasmid encoding the phosphotransferase gene *hygR* that confers resistance to the aminoglycoside hygromycin (Fig. 5a and Extended Data Fig. 5). *E. coli* cells equipped to record the *hygR* transcript were mixed with *E. coli* harboring the mobile *hygR* plasmid to promote conjugation. Sequencing individual transconjugant colonies uniformly yielded measurable editing for both Rptrs (56–90% for *hygR* Rptr1, 12–45% for *hygR* Rptr3), while sequencing unconjugated recipient colonies or transconjugant colonies receiving a scrambled tracrRNA yielded negligible editing (Fig. 5b and Extended Data Fig. 5). We speculate that the range of editing reflects the different timing of conjugation across the population. Tracking the spread of mobile genetic elements in a population therefore represents one application of TIGER that could reveal routes in which the mobile elements disseminated but were lost after further dissemination or failed to be maintained.

### Recording infection-induced sRNAs in *Salmonella*

The second broad area of application involves recording the expression of RNAs associated with key cellular events. These events could represent cellular differentiation processes or cellular programs induced by transient environmental conditions. In each case, the past event would no longer be detectable. As a proof-of-concept, we applied TIGER to record programmed expression changes within the food-borne, facultative intracellular pathogen *Salmonella* Typhimurium that are triggered during infection of human epithelial cells^44,45^. Previous work demonstrated that two sRNAs, PinT and OmrB, were upregulated in intracellular *Salmonella*^46^. PinT and OmrB are key posttranscriptional regulators of *Salmonella* virulence programs^47^ and enterobacteriaceae cell surface remodeling and motility^48,49^, respectively. These sRNAs represented an opportunity to demonstrate the ability of TIGER to record these key pathogenic events in a multiplexed manner.

**Extended Data Fig. 6:**
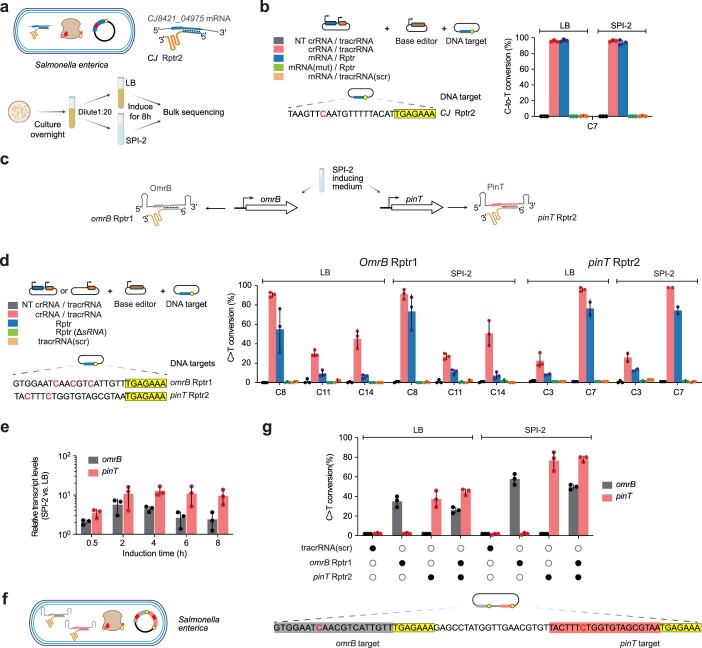
Bulk sequencing of recorded sRNAs OmrB and PinT in *Salmonella*. (**a**) Experimental setup for recording heterologous *CJ8421_04975* transcript in *Salmonella* under *in vitro* conditions simulating intracellular growth (SPI-2) or extracellular growth in rich medium (LB). (**b**) Bulk sequencing following the setup in a using a low-copy recording plasmid (~5 copies/cell). (**c**) Recording the OmrB and PinT sRNAs induced by SPI-2 inducing medium. (**d**) Bulk sequencing following the setup in a, only using either indicated *omrB* and *pinT* Rptrs and the corresponding DNA target. Δ*sRNA*: recording conducted in a strain in which the corresponding sRNA gene was deleted. Expression of the crRNA controls was driven by a constitutive promoter J23119, where efficient editing is expected even in the absence of sRNA induction. (**e**) Expression of OmrB and PinT in SPI-2 inducing medium versus LB medium as determined by RT-qPCR. The times indicate how long the cells were cultured in this medium before RNA isolation. (**f**) Experimental setup to simultaneously record OmrB and PinT. The setup follows that shown in **a** and **c**, only a medium-copy recording plasmid (~20 copies/cell) was used. (**g**) Co-recording of OmrB and PinT in *Salmonella* following the setup in **e**. Solid dot, presence of Rptr; Hollow dot, absence of Rptr. Values in **b**, **d**, **f** and **g** represent the mean and standard deviation of independent experiments starting from three separate colonies. Source data

After initially demonstrating that TIGER can record a heterologous transcript in *Salmonella* (Extended Data Fig. 6a,b), we turned to recording PinT and OmrB. Despite their small sizes (80 nts for PinT, 84 nts for OmrB), expression of either sRNA was successfully recorded under in vitro conditions that simulate an intracellular environment^50^ (Extended Data Fig. 6c,d). Editing was also lost when either the sRNA gene was deleted or a scrambled tracrRNA was used (Extended Data Fig. 6d). Under these growth conditions, the sRNAs were upregulated between two- and 14-fold (Extended Data Fig. 6e), in line with previous findings^46^. This upregulation yielded a sufficient boost in editing for state-specific recording when the DNA targets were placed together on a medium-copy plasmid (Extended Data Fig. 6f,g).

**Extended Data Fig. 7:**
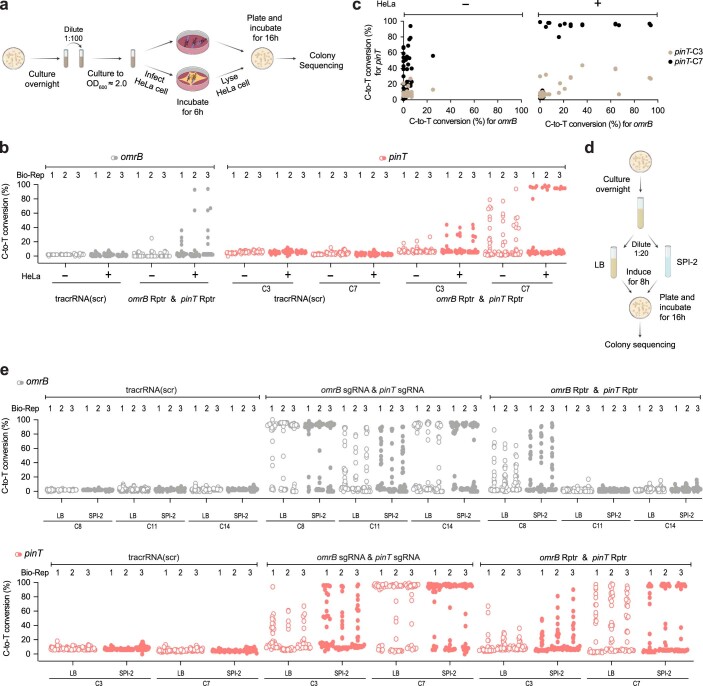
Recording of sRNAs OmrB and PinT in *Salmonella* colonies under different growth conditions. (**a**) Experimental setup for infection conditions. (**b**) Recording of OmrB and PinT in individual colonies following the setup in **a**. The same values were used in Fig. 5f. (**c**) Co-recording of OmrB and PinT in individual colonies following the setup in **a**. Values are plotted for two different positions within the *pinT* DNA target. The dots comprise measurements from all three biological replicates shown in **b**. The same values were used in Fig. 5g. (**d**) Experimental setup for *in vitro* growth conditions in non-inducing medium (LB) or inducing medium (SPI-2). (**e**) Recording of OmrB and PinT in individual colonies following the setup in **d**. For all experimental setups, *Salmonella* was equipped with the same recording machinery as in Extended Data Fig. 6f with a medium-copy recording plasmid. Each dot in **b** and **e** represents one sequenced colony, with clusters of dots representing one of three biological replicates starting from separate colonies. Source data

We then applied TIGER to simultaneously record PinT and OmrB as part of host cell invasion (Fig. 5c). TIGER-equipped *Salmonella* were used to infect a HeLa cell culture, while intracellular bacteria were recovered 6 hours postinfection and plated (Fig. 5d,e). *Salmonella* cells exposed to cell culture media alone served as controls. Colony sequencing revealed intermediate recording of *pinT* expression in the control condition (Fig. 5f and Extended Data Fig. 7a,b), in line with low-level expression of this sRNA by extracellular bacteria^45^. However, editing was elevated in *Salmonella* with an infection history despite the formed colonies having lost all memory of infecting a host (Fig. 5f and Extended Data Fig. 7a,b). Editing frequencies were also bimodal, with colonies exhibiting either no editing or intermediate to complete editing (Fig. 5f and Extended Data Fig. 7a,b). Multiplexed recording also indicated coexpression of PinT and OmrB in individual cells (Fig. 5g and Extended Data Fig. 7c). Bimodal expression has been associated with the regulators and targets of PinT and OmrB^46,50–52^. However, an expressed single-guide RNA (sgRNA) also yielded some unedited cells under inducing conditions in vitro (Extended Data Fig. 7d,e), suggesting that the recording machinery may be shut off in a portion of the population. TIGER thus can record infection-induced sRNAs in an intracellular pathogen, paving the way to interrogate single-cell responses over the course of an infection.

## Discussion

Here, we leveraged Rptrs to record expressed RNAs in single living cells through an approach we term TIGER. TIGER relies on the conversion of an RNA of interest into a gRNA, directing DNA editing by Cas9. Recent work demonstrated this conversion in *E. coli*^23,25^, in one example showing that the presence of an RNA of interest could be tied to GFP expression through CRISPR activation^25^. As the GFP signal would disappear along with the transcript, the approach remains limited to outputting the current state of a cell. By incorporating base editing to generate permanent DNA edits, we gained the ability to output past states. We further demonstrated that TIGER could record multiple selected transcripts, provide a quantitative read out of relative transcript levels, detect SNPs, determine the strength or duration of expression and capture single-cell phenomena, providing a detailed read out of a cell’s transcriptional history.

These capabilities contrast with the expanding set of methods to record transcriptional histories. Most of these methods rely on promoters to drive expression of the recording machinery^29–31,53–55^. While such approaches can capture different environmental signals and serve as a proxy for transcription of an RNA of interest, they cannot capture posttranscriptional regulation often implicated in sensory processes and may require long promoter regions to recapitulate natural regulation. Furthermore, these approaches would fail to record RNA viruses and would be difficult to detect any DNA invader unless the surrogate promoter is only expressed in the presence of the invader (for example, using the T7 promoter to sense the presence of T7 phage). Other recording methods that capture DNA could be used^29,56,57^, although they cannot be programmed to detect specific sequences. Beyond these methods, only one previous method can record RNA directly: an approach called RECORD-seq^19,58^. This method records the transcriptome through the random integration of RNA fragments as spacers in a CRISPR array, allowing quantitative recording of RNAs encoded throughout the genome and inference of the order of multiple stimuli based on spacer order. However, the combination of low acquisition frequencies and a large transcriptome means that massive populations of cells must be sequenced to infer the recorded RNAs, masking any single-cell phenomena. TIGER remains the only method that can selectively record individual RNAs at the single-cell level, offering a potential complement to RECORD-seq when aiming to record a smaller, defined set of transcripts.

By connecting past transcriptional events to current observations, TIGER has the potential to help answer outstanding scientific questions and enable new technological capabilities. Building on its use in different bacteria, TIGER could be applied to follow the spread of mobile genetic elements and their impact on a mixed microbial population, delineate the path of bacterial pathogens transiting through their host, or create sentinel cells that indirectly record healthy and diseased states in the human body and other environments. As the fundamental mechanisms of TIGER may extend to eukaryotic cells, we would further envision its use to track the trajectories of individual cells as part of cellular differentiation, RNA editing events in health and disease, or the development of chemoresistance in tumors or which host cells are infected but not killed by an invading pathogen. For all of these applications, TIGER is positioned to record different classes of RNAs, including protein-encoding transcripts, regulatory noncoding RNAs (for example, sRNAs, long noncoding RNAs), edited RNAs and, if applied to eukaryotic cells, mRNA splice variants.

In the process of characterizing and using TIGER, we identified key design considerations and areas for further improvement. Successful design required a compatible Rptr target with an upstream sequence harboring C in the editing window. The selected Rptr target should not contain a PAM to prevent editing of the transcript’s genomic site, although use of a Cas9 nuclease with a strict PAM (for example, Sth1Cas9) greatly reduces the likelihood of this scenario. While this design places some constraints on the region of a transcript that can be sensed, we showed that guide–target mismatches could introduce the necessary C or enhance editing with the preferred TC motif. Additionally, the design did not require a flanking PAM, as this could be readily incorporated as part of the DNA recording target. We further found that the copy number of the DNA target was an important design consideration, as higher copy numbers reduced editing frequencies but improved transcript quantification. Increasing the number of sensed RNAs through multiplexed recording also reduced editing frequencies, although this may be countered by relieving bottlenecks in the recording process (for example, increasing base editor expression). Finally, we found that sensing a transcript reduced its levels and subsequent translation for protein-coding RNAs. While this effect is expected as one form of retroactivity^59^, we showed that tuning Rptr expression could reduce this effect without compromising recording. Even then, this impact should be considered in case the act of recording meaningfully perturbs the associated cellular pathways. Overall, with further improvements, TIGER is poised to open new avenues for incorporating past transcriptional events into current observations.

## Methods

### Strains, oligonucleotides and plasmids

*E. coli* strain TOP10 was used for plasmid cloning. *E. coli* strains MG1655 and BW25113, and *Salmonella enterica* serovar Typhimurium strains were used for Rptr-based recording. All primers and gBlocks used in this work were ordered from Integrated DNA Technologies. NEBuilder HiFi DNA Assembly Master Mix (New England Biolabs, catalog no. E2621) was used for plasmid construction by Gibson assembly. Q5 site-directed mutagenesis kit (New England Biolabs, catalog no. E0554S) was used for small insertion and nucleotide substitution. Unless otherwise specified, all nucleases used were expressed in plasmids with chloramphenicol (Cm) selective marker and p15A origin-of-replication (roughly 15 copies per cell). All tracrRNA-crRNA, Rptr or Rptr-mRNA plasmids were expressed in plasmids with ampicillin (Amp) selective marker and pUC origin-of-replication (roughly 700 copies per cell), and all recording plasmids were expressed in plasmids with kanamycin (Kan) selective marker and pSC101 origin-of-replication (roughly five copies per cell). In some scenarios, for example, recording the upregulated sensed RNA responding to a certain stimulus, the background expression and induced expression of this RNA cannot be differentiated due to oversaturated editing with the low-copy recording plasmid. For better differentiation, the Rptr plasmid was placed in a low-copy plasmid (pSC101 ori, roughly five copies per cell) and the DNA target was placed in a medium-copy plasmid (pBR322 ori, roughly 20 copies per cell).

To construct the Sth1Cas9–D9A nickase (Sth1Cas9n) cytosine base editor plasmid, Sth1Cas9n coding sequence was PCR amplified from plasmid pCBS2148 and subjected to Gibson assembly with a PCR-linearized version of the vector pCBS1489. The resulting plasmid pCBS2149 was transformed and maintained in a low mutation strain MDS42 Meta LowMut (C-6786-10K, Scarab Genomics, LLC.). The consensus PAM (5′-NNAGAAW-3′, W = A, T) was used for Sth1Cas9 (refs. 27, 28,60). To construct recording plasmids, PAM-flanked target sequences were inserted directly upstream of the constitutive OR2-OR1 promoter with the template of pCB705 for low-copy editing plasmids (roughly five copies per cell) or directly downstream of AmpR terminator with the template of pCBS2895 for medium-copy editing plasmids (roughly 20 copies per cell) using Q5 site-directed mutagenesis. A λ-RED and FLP/FRT-mediated recombination system was used to construct *E. coli* Δ*xylA* or Δ*xylF* strains, and *S. enterica* Δ*pinT* or Δ*omrB* strains^61^. The *E. coli* Δ*rnc* strain was constructed using the ScCas9n cytosine base editor to introduce a premature stop codon into *rnc*. Gibson assembly was used to replace the Cm selective marker with hygromycin B (Hyg) selective marker in the F^+^ plasmid pDSW1728 for *E. coli* conjugation assay. For multiplexed recording of *xylF-xylA* and *xylA-rhaB*, *xylF* Rptr1_*xylA* Rptr2_*xylF* Rptr2 _*xylA* Rptr3 or *xylA* Rptr3_*rhaB* Rptr4 were cloned in tandem into the high-copy plasmid with a pUC origin-of-replication, respectively. Their corresponding PAM-flanked target sequences were also cloned in tandem into the recording plasmid. Four different combinations of PAM-flanked target sequences of *xylA* and *xylF* with different orientations and spacing intervals were constructed for multiplexed recording of *xylA* and *xylF*. See Supplementary Table 1 for detailed information and links to annotated plasmid maps.

### Media conditions

Super optimal broth with catabolite repression (SOC) medium containing 0.5% (w/v) yeast extract, 2% (w/v) tryptone, 10 mM NaCl, 2.5 mM KCl, 20 mM MgCl_2_ and 20 mM d-glucose was used to recover *E. coli* and *Salmonella* cells following transformation. Luria-Bertani (LB) medium containing 0.5% (w/v) yeast extract, 1% (w/v) tryptone and 0.5% (w/v) NaCl supplemented with antibiotics at appropriate concentrations were used to grow *E. coli* and *Salmonella* strains. 2% (w/v) agarose was added to made the corresponding LB plates. SPI-2 inducing medium (pH 5.8) containing 170 mM 2-(*N*-morpholino)ethanesulfonic acid, 5 mM KCl, 7.5 mM (NH_4_)_2_SO_4_, 0.5 mM K_2_SO_4_, 1 mM KH_2_PO_4_, 8 µM MgCl_2_, 38 mM glycerol and 0.1% (w/v) Bacto Casamino Acids (ThermoFisher Scientific, catalog no. 223050) was used for *Salmonella* in vitro assay. DMEM (Gibco) containing 4 mM l-glutamine, 4,500 mg l^−1^ glucose, 1 mM sodium pyruvate and 1,500 mg l^−1^ sodium bicarbonate, and additionally supplemented with 10% (w/v) fetal calf serum (Biochrom), 1% (w/v) sodium pyruvate and antibiotics at appropriate concentrations was used to grow HeLa cells for *Salmonella* infection. Antibiotics were added at final concentrations of 100 µg ml^−1^ for Amp, 34 µg ml^−1^ for Cm, 50 µg ml^−1^ for Kan, 100 µg ml^−1^ for Hyg and 10 or 50 µg ml^−1^ gentamicin (Gm) for *E. coli and S*. Typhimurium. Final concentrations of 1 mM for isopropyl-β-d-thiogalactoside (IPTG) and 20 mM for l-arabinose were used to induce the Sth1Cas9n base editor.

### Design of Rptrs and DNA targets

Rptrs were designed as previously described with minor modifications^23^. The antirepeat region of the native tracrRNA was replaced with the sequences complementary to the sensed RNAs and extra nucleotides were introduced into the antirepeat region to form a bulge, which is necessary to maintain Cas9 activity^62^. The regions in sensed RNAs bound by Rptrs were called Rptr targets. The total length of the Rptr target was 36 nts for Sth1Cas9n. The 20-nt sequences upstream of these Rptr target regions were treated as guides. The guides contain at least one C positioned in a window of the fifth to the tenth nucleotide counting from the Rptr target-distal end, which corresponds to the optimal editing window in the DNA target previously reported for the Sth1Cas9n base editor^63^. In cases where a C is not present in the optimal editing window of the equivalent target DNA, it is additionally introduced into the editing window in the recording plasmid. It is also recommended to include a T upstream of the editable C for better editing performance^26^. See Extended Data Fig. 1a for the design scheme.

### Electroporation

*E. coli* strains MG1655 and BW25113, and *S. enterica* serovar Typhimurium strains were streaked onto LB plates from cryostocks for overnight growth at 37 °C. Colonies were inoculated into LB liquid medium for overnight growth at 220 r.p.m. and 37 °C. The overnight cultures were back-diluted 1:50 into 50 ml of fresh LB liquid medium for 1.5 h growth at 220 r.p.m. and 37 °C. Cells were gathered and washed twice with 20 ml of 10% glycerol by centrifugation for 3 min at 4 °C and 4,500*g*. The cell pellets were resuspended with 1 ml of 10% glycerol, then transferred into 1.5 ml Eppendorf tubes and centrifuged for 1 min at 4 °C and 15,000*g*. The pellets were resuspended with 1 ml of 10% glycerol and split into 40-µl aliquots. Then 50–100 ng of Sth1Cas9n base editor, Rptr and recording plasmids were added to the cell suspensions. Electroporation was performed in a 1-mm gap cuvette (Cell Projects, catalog no. EP-101) at 1.8 kV, 200 Ω and 25 μF using Bio-Rad Gene Pulser Xcell. The cells were transferred into 500 µl of SOC medium and recovered for 1 h at 220 r.p.m. and 37 °C. 20 µl of recovered cultures for *E. coli* transformation and 150 µl for *Salmonella* transformation were spread on LB + Cm + Amp + Kan plates for overnight growth at 37 °C.

### Plasmid clearance assay in *E. coli*

Plasmid clearance in *E. coli* was conducted as previously described with slight modifications^64^. Briefly, 40 fmol of Rptr and scrambled tracrRNA plasmids were electroporated into *E. coli* BW25113 containing WT Sth1Cas9 and targeted plasmids. Transformed cells were recovered in SOC medium for 1 h at 37 °C and diluted 1:1,000 into LB + Cm + Kan liquid medium to maintain the Cas9 and targeted plasmids. Cultures were then grown overnight at 220 r.p.m. and 37 °C. Serial dilutions of the overnight cultures were spotted onto LB + Cm + Amp + Kan plates for overnight incubation at 37 °C. Colonies from countable spots were used to calculate the transformation fold-change with colony-forming units (CFUs) from scrambled tracrRNA as a baseline.

### Sth1Cas9n base editor editing window establishment

To identify the editing window for Sth1Cas9n base editor, three sgRNAs were designed with poly Cs located in different positions of the guide sequence. Colonies of *E. coli* containing the recording machinery were inoculated into LB + Cm + Amp + Kan liquid medium. Cultures were then grown overnight at 220 r.p.m. and 37 °C. The overnight cultures were back-diluted 1:20 into 5 ml of LB + Cm + Amp + Kan + IPTG + l-arabinose liquid medium for 8 h induction at 220 r.p.m. and 37 °C. Then 2 ml of induced cultures were collected for plasmid extraction using ZR Plasmid Miniprep-Classic kit (Zymo Research, catalog no. D4016). Next, 50 ng of the extracted plasmid was used as a template to amplify the edited region using primers CJpr0001 and CJpr0002. The PCR products were purified with the DNA Clean & Concentrator-5 kit (Zymo Research, catalog no. D4013) and then sent for Sanger sequencing with primer CJpr0001. The web tool EditR v.1.0.10 (https://moriaritylab.shinyapps.io/editr_v10/) was used to evaluate the editing efficiency^65^.

### Heterologous transcript recording

Bulk sequencing of the recording of *CJ8421_04975*, *degfp* and *dctA* transcripts followed the procedures in ‘Sth1Cas9n base editor editing window establishment’. In addition to the bulk sequencing of induced cultures, the 8 h-induced cultures for *CJ8421_04975* Rptr1 and Rptr2, and *degfp* Rptr2 were 1:5,000 diluted into 1× phosphate-buffered saline (PBS) before plating on LB + Cm + Amp + Kan plates for overnight growth at 37 °C. Then 20 random colonies were picked to check the editing efficiency by PCR amplifying the edited region using primers CJpr0001 and CJpr0002.

The 8 h-induced cultures for *CJ8421_04975* Rptr1 and Rptr2 were also subjected to single-cell sequencing as previously described with slight modifications^9^. The induced cultures were sampled and washed in 1× PBS twice, then diluted 1:120 into 1× PBS and sorted with an BD FACSAria III (70-µm nozzle, single-cell precision) into 96-well plates (Brand, catalog no. 781368) prefilled with 2.6 µl lysis buffer per well. The lysis buffer was assembled with 0.26 µl of 10× Lysis buffer (Takara, catalog no. 635013), 0.03 µl of RNase Inhibitor (100 U µl^−1^, Takara, catalog no. 2313A), 0.26 µl of 10× PBS, 0.1 µl of Lysozyme (50 U µl^−1^, Epicentre, catalog no. R1804M), 0.26 µl DTT (100 mM final concentration, Invitrogen), 0.026 µl EDTA (0.5 mM final concentration, Invitrogen) and 1.95 µl of nuclease-free water to a final volume of 2.6 µl. The sorted cells were then sonicated for 10 s (Sonorex Digitec DT 52, Bendelin) before subjected to amplification by adding 25 µl OneTaq PCR mixture (New England Biolabs, catalog no. M0482) containing primers CJpr2349 and CJpr2350. To obtain sufficient yield, a second PCR amplification was carried out with the first PCR product as the template. The PCR products for colony sequencing and single-cell sequencing were purified with the DNA Clean & Concentrator-5 kit (Zymo Research, catalog no. D4013) and then sent for Sanger sequencing with primer CJpr0001 for colony sequencing and CJpr2349 for single-cell sequencing. The web tool EditR v.1.0.10 was used to evaluate the editing efficiency^65^.

### Impact of plasmid copy number on editing dynamic range

*degfp* mRNA, *degfp* Rptr1 and its associated target were used to evaluate the impact of plasmid copy number of those elements on recording efficiency. Those three elements were cloned in low, medium or high-copy plasmids (roughly 5, 20 and 700 copies per cell, respectively) (Fig. 2f). The editing dynamic range was evaluated by bulk sequencing following the procedures in the method part ‘Sth1Cas9n base editor editing window establishment’ with slight modifications. The induction was performed for 7 h. Different primer pairs were used when amplifying and sequencing the edited plasmids with different copy numbers. For PCR amplification, primers CJpr0001 and CJpr0002 were used for the low-copy target, CJpr1376 and CJpr0299 for the medium-copy target, CJpr0298 and CJpr0299 for the high-copy target, respectively. The purified PCR products were then sent for Sanger sequencing with primer CJpr0001 for the low-copy target, CJpr1376 for the medium-copy target and CJpr0298 for the high-copy target.

### Quantitative recording

Plasmids encoding deGFP driven by different synthetic constitutive promoters in the J23100 series (J23119, J23100, J23116 and J23103) along with the base editor, and their corresponding target plasmids were transformed into *E. coli* MG1655-derived strain CB414. For the flow cytometry analysis, after 3 h induction, cultures were sampled and diluted 1:100 into 1× PBS and analyzed on an Accuri C6 flow cytometer with C6 sampler plate loader (Becton Dickinson) equipped with CFlow plate sampler, a 488-nm laser and a 530 ± 15-nm bandpass filter. Lower cutoff values of 11,500 and 500 were used for forward scatter (FSC-H) and side scatter (SSC-H), respectively. At least 50,000 gated events were collected for each measurement. Bulk sequencing, colony sequencing and single-cell sequencing were conducted as described in the section Heterologous transcript recording with slight modifications. For bulk sequencing, the induction was performed for 7 h. For bulk sequencing and colony sequencing, primers CJpr1376 and CJpr0299 were used to amplify the edited regions, and CJpr1376 was used for sequencing. For single-cell sequencing, primers CJpr2355 and CJpr2356 were used to amplify the edited regions, and CJpr2355 was used for sequencing.

### Essential gene recording

Three designed Rptrs targeting different regions of an essential gene *lpxC* were each cloned into a high-copy plasmid. Their targets were cloned in a low-copy plasmid, respectively. The base editor, Rptrs and their targets were transformed into *E. coli* MG1655-derived strain CB414. Bulk sequencing of the recording of the *lpxC* transcript followed the procedures in the section Sth1Cas9n base editor editing window establishment.

To evaluate the impact of *lpxC* Rptrs on growth, a transformation interference assay and a liquid growth assay were performed. For the transformation interference assay, 40 fmol of Rptr plasmid was electroporated into *E. coli* MG1655-derived strain CB414 with or without the base editor. Transformed cells were recovered in SOC medium for 1 h at 37 °C and serial dilutions of the recovered cultures were spotted onto LB + Amp plates for cultures without the base editor or LB + Cm + Amp + IPTG + l-arabinose plates for cultures with the base editor for overnight incubation at 37 °C. Colonies from countable spots were used to calculate the transformation fold-change with CFUs from scrambled tracrRNA as a baseline.

For the liquid growth assay, *lpxC* Rptrs together with or without base editor plasmids were transformed into *E. coli* MG1655-derived strain CB414, and a scrambled tracrRNA served as the negative control. Colonies of *E. coli* transformants were inoculated into LB liquid media supplemented with appropriate antibiotics. Cultures were then grown overnight at 220 r.p.m. and 37 °C. The overnight cultures were back-diluted 1:100 into a 96-well flat-bottom plate (Thermo Scientific, catalog no. 167008) prefilled with 120 µl of LB liquid media supplemented with appropriate antibiotics. For the cultures containing the base editor, IPTG and l-arabinose were added to induce the base editor. All cultures were adjusted to have the same initial optical density (OD_600_). The growth assay was performed by measuring the optical density on a Synergy Neo2 plate reader (BioTek). Time courses were run for 12 h at 37 °C with an interval of 5 min between reads.

### Impact of Rptrs on *degfp* transcript and protein levels

*degfp* mRNA driven by J23119 promoter and *degfp* Rptr1 driven by OR2-OR1 promoter were cloned in a low-copy plasmid. The resulting plasmids alone or together with the base editor were transformed into *E. coli* MG1655-derived strain CB414. Colonies were inoculated into 5 ml of LB liquid medium supplemented with appropriate antibiotics. Cultures were then grown overnight at 220 r.p.m. and 37 °C. The overnight cultures were back-diluted 1:20 into LB liquid medium supplemented with appropriate antibiotics and/or IPTG and l-arabinose when the base editor was present.

The cultures were incubated for 7 h at 220 r.p.m. and 37 °C. Then the cultures were collected and extracted using Direct-zol RNA Miniprep Plus kit (Zymo research, catalog no. R2072) with residual genomic DNA removed by on-column DNase I treatment. Primer amplification efficiency for *degfp* and the reference gene *ihfB* was evaluated using serially diluted DNA templates amplified from the plasmid or *E. coli* MG1655 genome. The extracted RNAs were quantitatively determined using the iTaq Universal SYBR Green One-Step Kit (Bio-Rad, catalog no. 1725151) on a Bio-Rad CFX96 instrument. The 2^−ΔΔCT^ method^66,67^ was used to evaluate *rhaB* transcript levels with the housekeeping gene *ihfB* as a reference for gene expression normalization.

A liquid growth assay was performed to evaluate the impact of Rptrs on the protein level of deGFP. The growth and fluorescence of back-diluted cultures were monitored on a Synergy Neo2 plate reader (BioTek) with an excitation filter of 485 nm and an emission filter of 528 nm. Time courses were run for 12 h at 37 °C with an interval of 5 min between reads. The fluorescence was normalized by dividing the fluorescence value by the optical density value (OD_600_).

### Rptrs driven by promoters with varying strengths

To evaluate the impact of Rptr expression on editing efficiency, *degfp* mRNA driven by J23119 promoter, *degfp* Rptr1 driven by different constitutive promoters (OR2-OR1, J23100, J23116 and J23103) and their associated target were cloned in a low-copy plasmid. The resulting plasmids along with the base editor were transformed into *E. coli* MG1655-derived strain CB414. The 7 h-induced cultures were subjected to bulk sequencing as described in the section Quantitative recording.

*degfp* expression was repressed when using a strong promoter, for example, OR2-OR1, to drive the Rptr expression. A growth-based assay was used to evaluate whether the Rptr driven by weaker promoters (J23100, J23116 and J23103) could mitigate the repression. Colonies of *E. coli* transformants equipped with TIGER were inoculated into LB + Cm + Kan liquid medium. Cultures were then grown overnight at 220 r.p.m. and 37 °C. The overnight cultures were back-diluted 1:100 into a 96-well flat-bottom plate (Thermo Scientific, catalog no. 167008) prefilled with 120 µl of LB + Cm + Kan + IPTG + l-arabinose liquid medium. The growth assay was performed by measuring fluorescence on a Synergy Neo2 plate reader (BioTek) with an excitation filter of 485 nm and an emission filter of 528 nm. Time courses were run for 12 h at 37 °C with an interval of 5 min between reads. The fluorescence was normalized by dividing the fluorescence value by the optical density value (OD_600_).

### Endogenous transcript recording

Colonies of *E. coli* equipped with the recording machinery were inoculated into LB + Cm + Amp + Kan liquid medium. Cultures were then grown overnight at 220 r.p.m. and 37 °C. The overnight cultures were back-diluted 1:20 into LB + Cm + Amp + Kan + IPTG + l-arabinose medium supplemented with d-xylose and/or l-rhamnose for 8 h induction at 220 r.p.m. and 37 °C. d-xylose and/or l-rhamnose were supplemented at a final concentration of 60 mM to induce endogenous genes *xylA*, *xylF* and/or *rhaB*, respectively. As control, the same amount of cultures was back-diluted 1:20 into LB + Cm + Amp + Kan + IPTG + l-arabinose liquid medium for 8 h induction. The amplification and sequencing steps followed those in the section Sth1Cas9n base editor editing window establishment.

### Transient recording

Colonies of *E. coli* strain CJ1163 were inoculated into 15 ml of LB + Cm + Amp + Kan liquid medium. Cultures were then grown overnight at 220 r.p.m. and 37 °C. The overnight cultures were centrifuged at 4,500*g* for 3 min, then resuspended into 50 ml of LB + Cm + Amp + Kan + IPTG + l-arabinose liquid medium for 1.5 h incubation at 220 r.p.m. and 37 °C. After 1.5 h incubation, 1 ml of cultures was harvested and resuspended in TRI Reagen for later RNA extraction. This was set as time point 0 h. The remaining cultures were collected, washed twice with 1× PBS and then suspended in 5 ml of LB + Cm + Amp + Kan + IPTG + l-arabinose liquid medium. The suspended cultures were back-diluted into 20 ml of LB + Cm + Amp + Kan + IPTG + l-arabinose + l-rhamnose liquid medium (final OD_600_ = 0.1) and subsequently exposed to l-rhamnose for 15 min, 1, 2, 4 and 8 h at 220 r.p.m. and 37 °C. As a negative control, the same volume of resuspended cultures was also back-diluted into 20 ml of l-rhamnose-free LB + Cm + Amp + Kan + IPTG + l-arabinose liquid medium for 8 h incubation. When l-rhamnose exposure was finished at time points 15 min, 1, 2 and 4 h, these cultures were harvested, washed twice with 1× PBS and then resuspended in 20 ml of l-rhamnose-free LB + Cm + Amp + Kan + IPTG + l-arabinose liquid medium to continue growth for 7.75, 7, 6 and 4 h, respectively, to fulfill the total 8 h induction time. Next 1–4 ml of culture was collected at time points 1, 2, 4 and 8 h regardless of medium replacement and resuspended in TRI Reagent for later RNA extraction. Samples were taken before medium replacement at time points 1, 2 and 4 h. When 8 h incubation finished, 2 ml of all cultures were collected for plasmid extraction using ZR Plasmid Miniprep-Classic kit (Zymo Research, catalog no. D4016). To further prove that DNA edits still remain even after the sensed RNAs disappears, the cells were cultured an additional 24, 48 and 96 h in LB medium without inducers and l-rhamnose after the 8 h exposure to LB + Cm + Amp + Kan + IPTG + l-arabinose + l-rhamnose liquid medium. Similarly, cultures were collected for plasmid extraction after each time point. Here, 50 ng of the extracted plasmid was used as a template to amplify the edited region using primers CJpr0001 and CJpr0002. The PCR products were purified with the DNA Clean & Concentrator-5 kit (Zymo Research, catalog no. D4013) and then sent for Sanger sequencing with primer CJpr0001. The web tool EditR v.1.0.10 was used to evaluate the editing efficiency^65^. The *rhaB* transcript level measurements by RT–qPCR referred to the Methods section Impact of Rptrs on *degfp* transcript and protein levels.

### Single-cell analysis

GFP expression driven by the *rhaB* promoter served as a read out to evaluate single-cell response to l-rhamnose. The *rhaB* target sequence was inserted downstream of the P*rhaB*-*gfp* expression cassette in the recording plasmid. The recording plasmid along with the base editor, and its corresponding Rptr plasmid were transformed into *E. coli* MG1655. Colonies of the corresponding strain CJ1215 were inoculated into 5 ml of LB + Cm + Amp + Kan liquid medium. Cultures were then grown overnight at 220 r.p.m. and 37 °C. The overnight cultures were back-diluted in a ratio of 1:20 into LB liquid medium + Cm + Amp + Kan + IPTG + l-arabinose, supplemented with various amounts of l-rhamnose (0, 10, 100, 333, 666, 1,000, 3,330, 6,660, 10,000 and 100,000 μM final concentration), and induced for 8 h at 220 r.p.m. and 37 °C. Single-cell responses to l-rhamnose were quantified by flow cytometry analysis as previously described with minor modifications^41^. After 8 h, cultures were sampled and diluted 1:100 into 1× PBS and analyzed on an Accuri C6 flow cytometer with C6 sampler plate loader (Becton Dickinson) equipped with CFlow plate sampler, a 488-nm laser and a 530 ± 15-nm bandpass filter. Lower cutoff values of 11,500 and 500 were used for forward scatter (FSC-H) and side scatter (SSC-H), respectively. At least 50,000 gated events were collected for each measurement. The gate for uninduced cells was set to a fluorescence intensity of 404, with >99% of events for the l-rhamnose-free control below the threshold. When generating the bubble plot (Extended Data Fig. 4c), each dot represented the mean of independent experiments starting from three separate colonies. The area of each dot scaled with the fraction of cells in that population.

The 8h-induced cultures with final l-rhamnose concentrations of 1,000 and 3,330 μM were 1:5,000 diluted into 1× PBS before plating on LB + Cm + Amp + Kan plates for overnight growth at 37 °C. Next 20 random colonies were picked to check the editing efficiency by PCR amplifying the edited region using primers CJpr1528 and CJpr1585. The PCR products were purified with the DNA Clean & Concentrator-5 kit (Zymo Research, catalog no. D4013) and then sent for Sanger sequencing with primer CJpr1585. The web tool EditR v.1.0.10 was used to evaluate the editing efficiency^65^.

The 8 h-induced cultures with a final l-rhamnose concentration of 3.33 mM were sampled and washed twice in 1× PBS, then diluted 1:120 into 1× PBS and analyzed using a BD FACSAria III (70-µm nozzle, single-cell precision). The gate for uninduced cells was set to a fluorescence intensity in the fluorescein isothiocyanate channel, where >99% of detected events in the l-rhamnose-free control sample were below the threshold. The gate for fully induced cells was set to a fluorescence intensity above 10^5^ in the fluorescein isothiocyanate channel. Next, 10,000 uninduced or fully induced cells were sorted into two separate tubes containing 200 μl of 1× PBS, respectively. The sorted cells were diluted and plated on LB + Cm + Amp + Kan plates for overnight growth at 37 °C. Then 20 random colonies were picked to check the editing efficiency by PCR amplifying the edited region using primers CJpr1528 and CJpr1585. The PCR products were purified with the DNA Clean & Concentrator-5 kit (Zymo Research, catalog no. D4013) and then sent for Sanger sequencing with primer CJpr1585. The web tool EditR v.1.0.10 was used to evaluate the editing efficiency^65^.

### Conjugation

*E. coli* K12 CGSC4401 containing plasmid pDSW1728-*hygR* served as the donor strain. *E. coli* BW25113 strains containing base editor, *hygR* Rptrs and recording plasmids served as the recipient strains. The donor strain and recipient strains were grown overnight at 220 r.p.m. and 37 °C in LB + Hyg and LB + Cm + Amp + Kan liquid media, respectively. The donor strain was 1:50 back-diluted into 100 ml LB + Hyg medium for 3 h growth at 220 r.p.m. and 37 °C. The recipient strains were 1:10 back-diluted into 25 ml LB + Cm + Amp + Kan + IPTG + l-arabinose medium for 3 h growth at 220 r.p.m. and 37 °C. After 3 h of growth, both donor strain and recipient strains were gathered, washed twice and then resuspended in 1× PBS. The mixtures of donor and recipient strains in a ratio of 10:1 were spotted onto LB + Cm + Amp + Kan + Hyg + IPTG + l-arabinose plates for 2–3 days of growth at 37 °C. As a negative control, the same amounts of recipient strains were spotted onto LB + Cm + Amp + Kan + IPTG + l-arabinose plates for 2–3 days of growth at 37 °C. Colony PCR was performed to amplify the editing region using primers CJpr0001 and CJpr0002. The PCR products were cleaned up using the DNA Clean & Concentrator-5 kit (Zymo Research, catalog no. D4013) and then sent for Sanger sequencing with primer CJpr0001. The web tool EditR v.1.0.10 was used to evaluate the editing efficiency^65^.

### Recording *Salmonella* sRNAs using SPI-2 inducing medium

Colonies were inoculated into LB + Cm + Amp + Kan liquid medium. Cultures were then grown overnight at 220 r.p.m. and 37 °C. The overnight cultures were back-diluted in a ratio of 1:20 into SPI-2 + Cm + Amp + Kan + IPTG + l-arabinose medium for 8 h induction at 220 r.p.m. and 37 °C. As control, the same volume of cultures was back-diluted in a ratio of 1:20 into LB + Cm + Amp + Kan + IPTG + l-arabinose medium for 8 h induction. The induced cultures were diluted 1:5,000 into 1× PBS before plating on LB + Amp plates for overnight growth at 37 °C. Next, 30 random colonies were picked to check the editing efficiency by PCR amplifying the edited region using primers CJpr1376 and CJpr0299. The PCR products were purified with the DNA Clean & Concentrator-5 kit (Zymo Research, catalog no. D4013) and then sent for Sanger sequencing with primer CJpr1376. The web tool EditR v.1.0.10 (ref. 65) was used to evaluate the editing efficiency.

### *Salmonella* infection assay

HeLa 229 (ATCC CCL-2.1) cell infection with *Salmonella* Typhimurium SL1344 was carried out as previously described with slight modifications^46,68^. Two days before infection, 2 × 10^5^ HeLa cells were seeded in 2 ml of DMEM (Gibco) + 10% fetal calf serum (Biochrom) + 1% sodium pyruvate (Gibco) (referred to as cell culture medium) in a six-well format. Three colonies of *Salmonella* were inoculated into 5 ml of LB + Amp + Kan + Cm liquid medium and grown overnight at 37 °C and 220 r.p.m. The overnight cultures were diluted 1:100 in 10 ml of fresh LB + Amp (100 µg ml^−1^) + Kan (50 µg ml^−1^) + Cm (34 µg ml^−1^) medium and grown aerobically to an OD_600_ of 2.0. Infection of HeLa cells was carried out by diluting the bacteria in cell culture medium + Amp + Kan + Cm, aspirating HeLa cell culture supernatants and adding the bacterial suspension to each well with a multiplicity of infection of 10. As a control, the same amount of *Salmonella* cells in cell culture medium + Amp + Kan + Cm was added to empty wells without HeLa cells. Immediately after addition of bacteria, the plates were centrifuged for 10 min at 250*g* at room temperature followed by 30 min incubation in 5% CO_2_ humidified atmosphere at 37 °C. As a control, the plates were incubated for 10 min at room temperature (without centrifugation) followed by 30 min in 5% CO_2_ humidified atmosphere at 37 °C. For the infected samples, medium was then replaced with cell culture medium + Amp + Kan + Cm + IPTG (1 mM) + l-arabinose (20 mM) + 50 μg ml^−1^ gentamicin to induce the TIGER machinery and kill extracellular bacteria. This was set as time point 0 h (*t*0). After a further 30 min incubation step, medium was again replaced with fresh cell culture medium + Amp + Kan + Cm + IPTG + l-arabinose + 10 μg ml^−1^ gentamicin, and incubated for the remainder of the experiment. For the control group, 2 µl of 1 M IPTG and 30 µl of 1.33 M l-arabinose were added directly to the bacterial cells in cell culture medium containing no gentamicin at *t*0. For plating of CFUs at the respective time points, the wells were washed twice with 1× DPBS (Gibco) and 2 ml of 0.1% Triton-X DPBS were added to selectively lyse the host cells. After 5 min at room temperature, the cells were scratched off the bottom of the wells, resuspended using a 1 ml tip and serially diluted before plating on LB + Amp plates for overnight growth at 37 °C. The rest of the cell lysates was kept at 4 °C for plasmid extraction and Sanger sequencing. Next, 30 random colonies from three independent biological replicates were picked to check the editing efficiency by PCR amplifying the edited region using primers CJpr1376 and CJpr0299. The PCR products were purified with the DNA Clean & Concentrator-5 kit (Zymo Research, catalog no. D4013) and then sent for Sanger sequencing with primer CJpr1376. The web tool EditR v.1.0.10 was used to evaluate the editing efficiency^65^.

### Statistical analyses

Statistical significance was calculated using a two-tailed Student’s *t*-test assuming unequal variance. Values were assumed to be normally distributed. Linear regression was used for correlation analysis for *gfp* quantitative recording and the impact of l-rhamnose induction time on editing efficiency. The threshold of significance for the calculated *P* values was set as 0.05.

### Reporting summary

Further information on research design is available in the Nature Portfolio Reporting Summary linked to this article.

## Online content

Any methods, additional references, Nature Portfolio reporting summaries, source data, extended data, supplementary information, acknowledgements, peer review information; details of author contributions and competing interests; and statements of data and code availability are available at 10.1038/s41587-022-01604-8.

## Supplementary information

Reporting Summary

Supplementary Table 1 List of strains and DNA constructs, including plasmids, gBlocks, amplified linear DNA and oligonucleotides.

## Data Availability

Selected plasmids used in this study are being made available from Addgene. Source data are provided for Figs. 1–5 and Extended Data Figs. 1 and 3–7.

## Source data

Source Data Fig. 1 Statistical source data.
Source Data Fig. 2 Statistical source data.
Source Data Fig. 3 Statistical source data.
Source Data Fig. 4 Statistical source data.
Source Data Fig. 5 Statistical source data.
Source Data Extended Data Fig. 1 Statistical source data.
Source Data Extended Data Fig. 3 Statistical source data.
Source Data Extended Data Fig. 4 Statistical source data.
Source Data Extended Data Fig. 5 Statistical source data.
Source Data Extended Data Fig. 6 Statistical source data.
Source Data Extended Data Fig. 7 Statistical source data.
