# Supplementary material for: RNA recording in single bacterial cells using reprogrammed tracrRNAs
Source: Nat Biotechnol. 2023 Jan 5;41(8):1107–16. doi: 10.1038/s41587-022-01604-8 (PMC7614944; doi:10.1038/s41587-022-01604-8)
Supplement: Supplementary file 1 — Reporting Summary [file 41587_2022_1604_MOESM1_ESM.pdf]

Corresponding author(s): Chase L. Beisel
Last updated by author(s): Nov 2, 2022

Reporting Summary

Nature Research wishes to improve the reproducibility of the work that we publish. This form provides structure for consistency and transparency in reporting. For further information on Nature Research policies, see our Editorial Policies and the Editorial Policy Checklist.

Statistics

For all statistical analyses, confirm that the following items are present in the figure legend, table legend, main text, or Methods section.

n/a	Confirmed	
☐	☒	The exact sample size (n) for each experimental group/condition, given as a discrete number and unit of measurement
☐	☒	A statement on whether measurements were taken from distinct samples or whether the same sample was measured repeatedly
☐	☒	The statistical test(s) used AND whether they are one- or two-sided<br>*Only common tests should be described solely by name; describe more complex techniques in the Methods section.*
☒	☐	A description of all covariates tested
☐	☒	A description of any assumptions or corrections, such as tests of normality and adjustment for multiple comparisons
☐	☒	A full description of the statistical parameters including central tendency (e.g. means) or other basic estimates (e.g. regression coefficient) AND variation (e.g. standard deviation) or associated estimates of uncertainty (e.g. confidence intervals)
☐	☒	For null hypothesis testing, the test statistic (e.g. F, t, r) with confidence intervals, effect sizes, degrees of freedom and P value noted<br>*Give P values as exact values whenever suitable.*
☒	☐	For Bayesian analysis, information on the choice of priors and Markov chain Monte Carlo settings
☒	☐	For hierarchical and complex designs, identification of the appropriate level for tests and full reporting of outcomes
☒	☐	Estimates of effect sizes (e.g. Cohen's d, Pearson's r), indicating how they were calculated

Our web collection on statistics for biologists contains articles on many of the points above.

Software and code

Policy information about availability of computer code

Data collection	Accuri C6 Plus flow cytometer software and BD FACSDiva 8.0.1
Data analysis	A web tool EditR 1.0.10 (https://moriaritylab.shinyapps.io/editr_v10/) was used to evaluate the editing efficiency.

For manuscripts utilizing custom algorithms or software that are central to the research but not yet described in published literature, software must be made available to editors and reviewers. We strongly encourage code deposition in a community repository (e.g. GitHub). See the Nature Research guidelines for submitting code & software for further information.

Data

Policy information about availability of data

All manuscripts must include a data availability statement. This statement should provide the following information, where applicable:
- Accession codes, unique identifiers, or web links for publicly available datasets
- A list of figures that have associated raw data
- A description of any restrictions on data availability

Selected plasmids used in this study are being made available from Addgene. Source data are provided for Figures 1-5 and Extended Data Figures 1-7.

Field-specific reporting

Please select the one below that is the best fit for your research. If you are not sure, read the appropriate sections before making your selection.

☒ Life sciences ☐ Behavioural & social sciences ☐ Ecological, evolutionary & environmental sciences

For a reference copy of the document with all sections, see nature.com/documents/nr-reporting-summary-flat.pdf

Life sciences study design

All studies must disclose on these points even when the disclosure is negative.

Sample size	At least three random colonies were picked as biological replication for each assay. The number of selected colonies were determined based on standards in the field for these types of experiments.
Data exclusions	Experiments were done with at least three replicates. All reported data were reproducible. Only data that were not reproducible because of errors in the experimental procedure were excluded.
Replication	Values in the figures represent the average of at least three independent experiments starting from separate colonies.
Randomization	Random colonies of transformants were picked as biological replicates. Other aspects of randomization (e.g., covariates) are not relevant.
Blinding	Blinding is not relevant to the study, as blinding is not normally included for bacterial and cell culture experiments.

Reporting for specific materials, systems and methods

We require information from authors about some types of materials, experimental systems and methods used in many studies. Here, indicate whether each material, system or method listed is relevant to your study. If you are not sure if a list item applies to your research, read the appropriate section before selecting a response.

Materials & experimental systems

n/a	Involved in the study	
☒	☐	Antibodies
☐	☒	Eukaryotic cell lines
☒	☐	Palaeontology and archaeology
☒	☐	Animals and other organisms
☒	☐	Human research participants
☒	☐	Clinical data
☒	☐	Dual use research of concern

Methods

n/a	Involved in the study	
☒	☐	ChIP-seq
☐	☒	Flow cytometry
☒	☐	MRI-based neuroimaging

Eukaryotic cell lines

Policy information about cell lines

Cell line source(s)	HeLa 229 (ATCC CCL-2.1) cell
Authentication	The cell lines were obtained directly from ATCC. No additional authentication was applied.
Mycoplasma contamination	The cell line was not tested for mycoplasma contamination.
Commonly misidentified lines (See ICLAC register)	No commonly misidentified cell lines were used in this study.

Flow Cytometry

Plots

Confirm that:

☒ The axis labels state the marker and fluorochrome used (e.g. CD4-FITC).

☒ The axis scales are clearly visible. Include numbers along axes only for bottom left plot of group (a 'group' is an analysis of identical markers).

☒ All plots are contour plots with outliers or pseudocolor plots.

☒ A numerical value for number of cells or percentage (with statistics) is provided.

Methodology

Sample preparation	Overnight E. coli cells were diluted into fresh medium followed by an 8h induction. Then the induced cultures were sampled and diluted 1:100 into 1X PBS before flow cytometry or Fluorescence-activated Cell Sorting (FACS) analysis. See Methods.
Instrument	Accuri C6 Plus flow cytometer with BD CSampler Plus (Becton Dickinson) and BD FACSAria™ III
Software	Accuri C6 Plus flow cytometer software and BD FACSDiva 8.0.1
Cell population abundance	Flow Cytometry was used for measuring the GFP expression of E. coli, no sorting was done. FACS was used to isolate single cells for single-cell DNA sequencing or sort low-fluorescent cells and high-fluorescent cells into different tubes for subsequent colony sequencing.
Gating strategy	For flow cytometry analysis, Lower cut-off values of 11,500 and 500 were used for forward scatter (FSC-H) and side scatter (SSC-H), respectively. For flow cytometry analysis, no figures were provided to exemplifying the gating strategy.<br>For FACS, A side scatter (SSC-A) vs. forward scatter (FSC-A) density plot was used to exclude doublets and debris to get the singlets. Fig. S2 provided the gating strategy for FACS. See Methods.

☒ Tick this box to confirm that a figure exemplifying the gating strategy is provided in the Supplementary Information.
